# Development and Validation of a HPTLC Method for the Estimation of Sumatriptan in Tablet Dosage Forms

**DOI:** 10.4103/0250-474X.49138

**Published:** 2008

**Authors:** C. R. Shah, B. N. Suhagia, N. J. Shah, R. R. Shah

**Affiliations:** Shri B. M. Shah College of Pharmaceutical Education and Research, Modasa-383 315, India; 1 L. M. College of Pharmacy, Ahmedabad-380 009, India; 2 Swaminarayan Sanskar Pharmacy College, Zundal-380 009, India

**Keywords:** HPTLC, sumatriptan, validation

## Abstract

A simple, precise, accurate and rapid high performance thin layer chromatographic method has been developed and validated for the estimation of sumatriptan in tablet dosage forms. The stationary phase used was precoated silica gel 60F254. The mobile phase used was a mixture of methanol:water:glacial acetic acid (4.0:8.0:0.1, v/v/v). The detection of spots was carried out at 230 nm. The method was validated in terms of linearity, accuracy, precision and specificity. The calibration curve was found to be linear between 200 to 800 ng/spot. The limit of detection and the limit of quantification for the sumatriptan were found to be 63.87 and 193.54 ng/spot, respectively. The proposed method can be successfully used to determine the drug content of marketed formulation.

Sumatriptan succinate (SUM) is described chemically as: 3-[2-(dimethylamino)ethyl]-N-methyl-indole-5-methanesulfonamide succinate^1^. SUM is a selective 5-hydroxytryptamine_1_ receptor subtype agonist. It is an antimigraine drug, structurally similar to serotonin^2^. Literature survey revealed that few HPLC methods in plasma^3^ and brain tissue^4^, HPLC-MS-MS^5^,^6^, HPLC-ECD^7^,^8^, HPLC-coulometric^9^, capillary LC-MS-MS^10^, HPLC-tandem MS-MS^11^, HPLC with fluorescence detection^12^ were reported for the determination of SUM. So far no HPTLC method has been reported for the estimation of sumatriptan in tablet dosage forms. In the present investigation an attempt has been made to develop accurate and precise HPTLC method for the estimation of sumatriptan in tablet dosage forms.

Sumatriptan standard was procured as a gift sample from Intas Pharmaceuticals Ltd., Ahmedabad. Silica gel 60F_254_ TLC plates (10×10 cm, layer thickness 0.2 mm, E. Merck, Mumbai) were used as a stationary phase. All chemicals and reagents used were of analytical grade. Tablets containing sumatriptan (25 mg) were purchased from local market Suminat (Sun Pharmaceuticals Ind. Ltd, Silvasa) and Sumitrex (Sun Pharmaceutical Ind. Ltd, Mumbai). A Camag HPTLC system comprising of Camag Linnomate V automatic sample applicator, Hamilton syringe (100 μl), Camag TLC Scanner 3, Camag WinCATS software, Camag Twin-trough chamber (10×10 cm) and ultrasonicator were used during study.

Sumatriptan (25 mg) was weighed accurately, dissolved and diluted with methanol to obtain the final concentration of 100 μg/ml of each drug. Twenty tablets were weighed accurately and ground to fine powder. Weight equivalent to 25 mg of sumatriptan was transferred to conical flask and mixed with methanol. The solution was sonicated for 15 min. The extracts were filtered through Whatmann filter paper No. 41 and residue was washed with methanol. The extracts and washing were pooled and transferred to a 250 ml volumetric flask and volume was made up to 250 ml with methanol to get 100 μg/ml of sumatriptan.

TLC plates were prewashed with methanol. Activation of plates was done in an oven at 50° for 5 min. The chromatographic conditions maintained were precoated silica gel 60F_254_ aluminum sheets (10×10 cm) as stationary phase, methanol:water:glacial acetic acid (4.0:8.0:0.1, v/v/v) as mobile phase, chamber and plate saturation time of 30 min, migration distance allowed was 72 mm, wavelength scanning was done at 230 nm keeping the slit dimension at 5×0.45 mm. A deuterium lamp provided the source of radiation. Three microlitres of standard solutions of sumatriptan were spotted and developed at constant temperature. Wavelength was selected by scanning standard solutions of drug over 200 nm to 400 nm wavelength. Sumatriptan shows maximum absorbance at 230 nm, therefore photometric measurements was performed at 230 nm in reflectance mode with Camag TLC scanner 3 using Win CATS software.

Aliquots of 2.0, 3.0, 4.0, 5.0, 6.0, 7.0, and 8.0 μl of standard solution of sumatriptan were applied on the TLC plate (100 μg/ml of drug). TLC plate was dried, developed and analyzed photometrically as described earlier. The standard calibration curve was generated using regression analysis with Microsoft Excel.

The developed method was validated in terms of linearity, accuracy, limit of detection, limit of quantification, intra-day and inter-day precision and repeatability of measurement as well as repeatability of sample application^13^,^14^.

Four microlitres of sample solutions of the marketed formulations were spotted on to the same plate followed by development scanning. The analysis was repeated in triplicate. The content of the drug was calculated from the peak areas recorded.

A solvent system that would give dense and compact spot with significant R_f_ value was desired for the quantification of sumatriptan in pharmaceutical formulations. The mobile phase consisting of methanol:water:glacial acetic acid (4.0:8.0:0.1, v/v/v) gave R_f_ value of 0.64±0.01 for sumatriptan (fig. 1). The linear regression data (n=5, Table 1) showed a good linear relationship over a concentration range of 200-800 ng/spot for sumatriptan. The limit of detection and limit of quantification for sumatriptan were found to be 63.87 and 193.54 ng/spot.

**Fig. 1:**
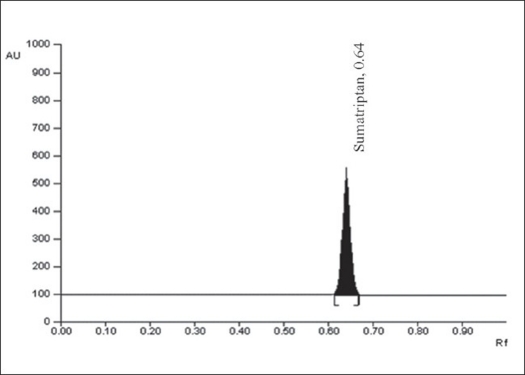
A typical HPTLC Chromatogram of Sumatriptan HPTLC chromatogram represents peak of sumatriptan at 0.64±0.01.

**TABLE 1:** METHOD VALIDATION PARAMETERS

Parameters	Values Sumatriptan
Linearity range (ng/spot)	200-800
Correlation coefficient (r)	0.9993
Regression equation (y=mx+c)	
Slope (m)	7.6279
Intercept (c)	-21.2
Limit of detection (LOD)	63.87 ng/spot
Limit of quantification (LOQ)	193.54 ng/spot
Precision (%CV)	
Repeatability of application (n=5)	1.31
Repeatability of measurement (n=5)	1.15

The intra-day precision was determined by analyzing standard solutions in the concentration range of 300 ng/spot to 600 ng/spot for 3 times on the same day while inter-day precision was determined by analyzing corresponding standards daily for 3 day over a period of one week. The intra-day and inter-day coefficients of variation are given in Table 2. Repeatability of sample application was assessed by spotting 4 μl of drug solution 5 times on a TLC plate followed by development of plate and recording the peak area for 5 spots. The % RSD for peak area values of sumatriptan was found to be 1.31. Repeatability of measurement of peak area was determined by spotting 4 μl of sumatriptan solution on a TLC plate and developing the plate. The separated spot was scanned five times without changing the position of the plate and % RSD for measurement of peak area of sumatriptan was found to be 1.15. To confirm the specificity of the proposed method, the solution of the formulation was spotted on the TLC plate, developed and scanned. It was observed that the excipients present in the formulation did not interfere with the peak of sumatriptan.

**TABLE 2:** PRECISION OF SUMATRIPTAN

Drug	Concentration (μg/spot)	Intra-day precision % RSD	Inter-day precision % RSD
Sumatriptan	3.0	0.729	1.402
25 (mg)	4.0	0.981	1.048
	5.0	0.876	0.911
	6.0	0.732	0.849

RSD is relative standard deviation.

Recovery studies of the drugs were carried out for the accuracy parameter. These studies were carried out at three levels i.e. multiple level recovery studies. Sample stock solution from tablet formulation of 100 μg/ml was prepared. Dilutions were made and recovery studies were performed. Per cent recovery was found to be within the limit. The assay value for the marketed formulation was found to be within the limit. The low RSD value indicated the suitability of the method for routine analysis of sumatriptan in pharmaceutical dosage forms. The developed HPTLC technique is simple, precise, specific and accurate and the statistical analysis proved that method is reproducible and selective for the analysis of sumatriptan in bulk drug and tablet formulations.
